# Common and Distinct Genetic Architecture of Blood Pressure in Relation to Coronary Artery and Abdominal Aortic Calcium

**DOI:** 10.5334/gh.1385

**Published:** 2025-01-13

**Authors:** Haozhang Huang, Huangtao Ruan, Xiaozhao Lu, Weipeng Zhang, Jin Liu

**Affiliations:** 1 Department of Cardiology, Guangdong Cardiovascular Institute, Guangdong Provincial People’s Hospital (Guangdong Academy of Medical Sciences), Southern Medical University, 510080, China; 2 Guangdong Provincial Key Laboratory of Coronary Heart Disease Prevention, Guangdong Provincial People’s Hospital, Guangdong Academy of Medical Sciences, Guangzhou, 510080, China

**Keywords:** Blood pressure, coronary artery calcium, abdominal aortic calcium, genetic architecture

## Background

Coronary artery calcium (CAC) and abdominal aortic calcium (AAC) are established as independent predictors for cardiovascular disease risk (1,2). CAC primarily indicates atherosclerotic intimal calcification, which influences coronary artery disease, whereas AAC involves both medial and intimal calcification, contributing to arterial stiffness, particularly in younger demographics (3,4). Vascular calcification presents a significant complication of hypertension. The distinct pathophysiological roles of CAC and AAC suggest that blood pressure may have differential and common genetic influences on these calcification processes.

## Methods

We utilized genome-wide association study (GWAS) data on systolic blood pressure (SBP) and diastolic blood pressure (DBP) sourced from the International Consortium of Blood Pressure, offering robust genetic insights by aggregating data from multiple cohorts (5). GWAS data for CAC were obtained from 28,655 individuals in the Heart and Aging Research in Genomic Epidemiology (CHARGE) consortium and associated cohorts (6). AAC data were derived from GWAS as part of the UK Biobank imaging study, involving 38,264 individuals of European ancestry (7). AAC was quantified using a standardized scoring approach based on visible calcification in the anterior and posterior aortic walls.

Genetic correlations between SBP, DBP, and both CAC and AAC were assessed using linkage disequilibrium score regression (LDSC) and two-sample Mendelian randomization (MR). We validated instrumental variables by aligning exposure and outcome effects and excluded single nucleotide polymorphisms (SNPs) with insufficient *F*-statistics. To mitigate potential reverse causation, Steiger filtering was applied. Each analysis employed the inverse variance–weighted (IVW) MR method with a random effect model, and we examined the *P* value for the intercept from MR Egger regression to check for horizontal pleiotropy.

Shared risk loci were identified through Heritability Estimation from Summary Statistics (ρ-HESS), cross-trait meta-analyses, and colocalization techniques. ρ-HESS provides local SNP-heritability and genetic correlation estimates. We conducted two cross-trait meta-analyses, including the multi-trait analysis of GWAS (MTAG) and cross phenotype association test (CPASSOC). Novel loci were characterized as independent SNPs without linkage disequilibrium (LD_ r_2 > 0.2 within 500-kb windows) with significant SNPs in the original single-trait GWAS. Core SNPs were prioritized as genome-wide significant (*P* < 5 × 10^–8^) in the cross-trait meta-analyses using both MTAG and CPASSOC and were in significant regions identified by ρ-HESS. A locus was considered colocalized if the probability for H4 (PPH4) exceeded 0.95. Genomes pathway enrichment analyses of the shared risk loci were performed. Data analyses were conducted using R version 4.3.1 (R Project for Statistical Computing, Vienna, Austria) and Python 2.7 (Python Software Foundation, Wilmington, USA).

## Results

**Figure 1:**
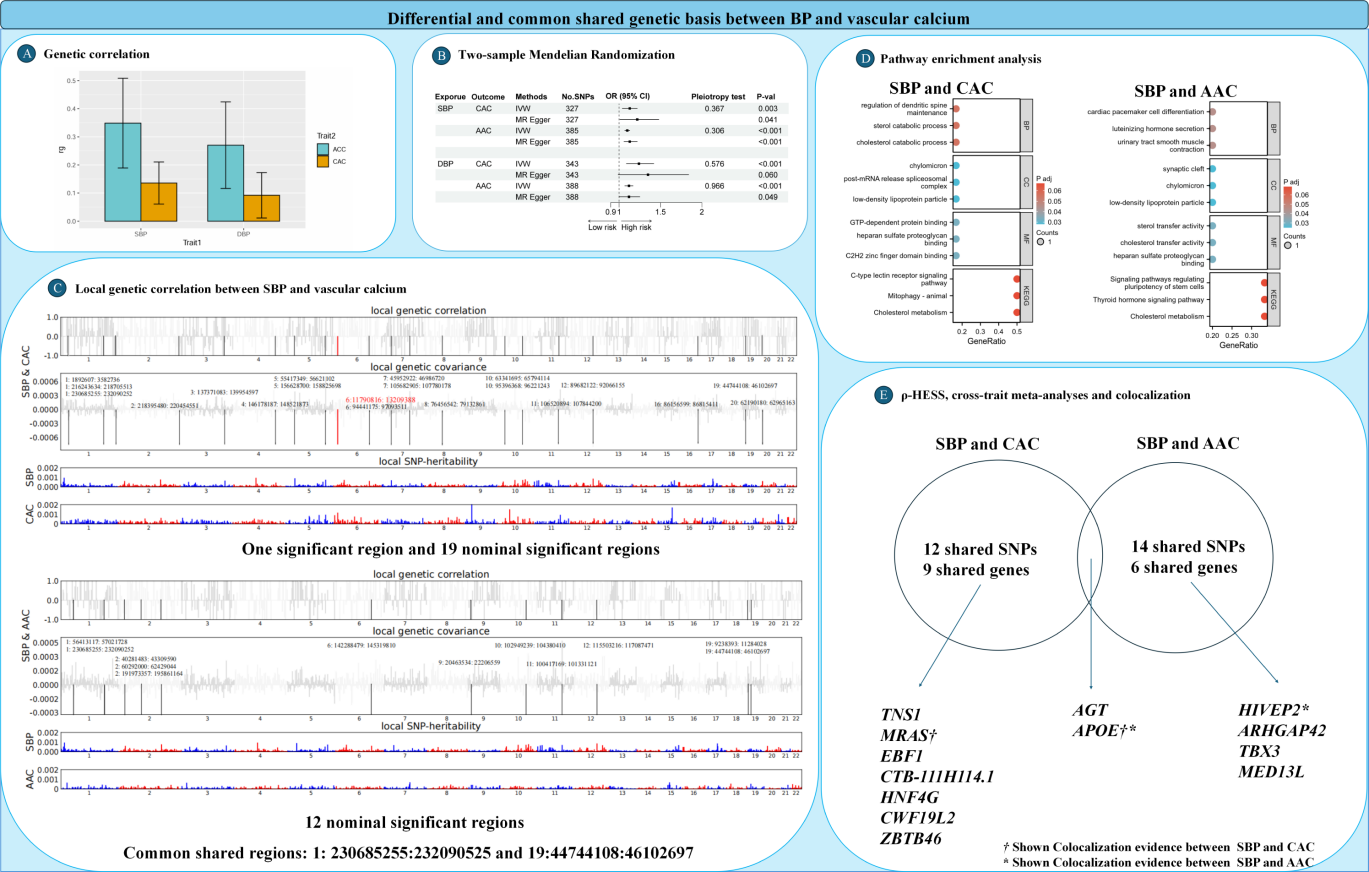
**Differential and common shared genetic basis between BP and vascular calcium. (A)** Genetic correlation between SBP and vascular calcium using LDSC. **(B)** Two-sample Mendelian randomization analyses between BP and vascular calcium. **(C)** Local genetic correlation between SBP and vascular calcium. The Manhattan plot showed the estimates of local genetic correlation and local genetic covariance between LTL and MDD, and local SNP heritability of LTL and MDD, respectively. Red and blue bars in ‘local genetic correlation’ and ‘local genetic covariance’ represent significant regions which shared SNP heritability, after multiple adjustments (*P* < 5E-08 in both local SNP heritability test, and *P* < 0.05/1703 in local genetic covariance test). Black bars showed nominal significant (*P* < 0.05). **(D)** Pathway enrichment analysis of shared SNPs of SBP and vascular calcium. **(E)** Significant pleiotropic SNPs identified by cross-trait meta-analyses and ρ-HESS.

The genetic correlation between SBP and CAC was 0.136, while the correlation between DBP and CAC was 0.092 (*P* < 0.001) (Figure 1A). For AAC, the genetic correlation with SBP was 0.349, and with DBP overall it was 0.270 (*P* < 0.001; Figure 1A). Two-sample MR analysis suggested a reliable causal effect of BP on vascular calcification (IVW-*P* < 0.05, pleiotropy test >0.05; Figure 1B).

After multiple corrections, a pronounced local correlation was identified in a single region on chromosome 6: 11790816-13209388 between SBP and CAC (*P* = 6.58 × 10^–6^) along with 19 nominally significant regions (Figure 1C). Between SBP and AAC, 12 nominally significant regions were identified, led by chromosome 11: 100417169-101331121 (*P* = 0.004). Common shared regions included chromosome 1: 230685255-232090252, and chromosome 19: 44744108-46102697.

The pathway enrichment analysis for SBP and CAC revealed significant associations with processes such as dendritic spine maintenance, sterol catabolic processes, and low-density lipoprotein particles. Key molecular functions include GTP-dependent protein binding and C_2_H_2_ zinc finger domain binding, with notable pathways like cholesterol metabolism showing enrichment. For SBP and AAC, the analysis highlighted pathways involving cardiac pacemaker cell differentiation and signaling pathways regulating pluripotency of stem cells. Molecular functions such as sterol transfer activity and heparan sulfate proteoglycan binding were also enriched, alongside critical pathways like thyroid hormone signaling and cholesterol metabolism (Figure 1D).

Twelve shared independent SNPs and nine genes were identified by ρ-HESS and cross-trait meta-analysis in the joint phenotype SBP-CAC (Figure 1E). Furthermore, two novel loci (rs34905952 located in *MRAS* and rs7412 located in *APOE*) shared with SBP-CAC showed evidence of colocalization (PPH4 > 0.95). Fourteen shared independent SNPs and six genes were identified in the joint phenotype SBP-AAC, with two novel loci (rs6570530 located in *HIVEP2* and rs7412 located in *APOE*) also showing evidence of colocalization (PPH4 > 0.95).

## Discussion

This study provides significant insights into the genetic underpinnings of vascular calcification in relation to blood pressure, highlighting distinct and overlapping genetic influences on CAC and AAC. We identified measurable genetic correlations between both SBP and DBP with CAC and AAC, emphasizing the complex interplay between BP regulation and vascular calcification. Our use of LDSC and MR underscores the causal relationship of blood pressure effects on vascular calcification.

Notably, we discovered pronounced local correlations and significant regions shared between SBP and CAC/AAC, including novel loci such as rs34905952 in *MRAS* (8), rs6570530 located in *HIVEP2* (9), and rs7412 in *APOE* (10), indicating potential targets for genetic investigation and therapeutic intervention. The differentiation between genetic influences on CAC and AAC suggests targeted approaches may be necessary for prevention and treatment tailored to specific types of vascular calcification. These findings extend our understanding of genetic architecture linking blood pressure with vascular calcifications, offering a basis for future research to unravel the complex genetic networks at play and identify potential biomarkers or targets for intervention in cardiovascular disease. And distinct and overlapping pathways underline the complex genetic interactions influencing blood pressure and vascular calcification, offering insights into targeted therapeutic strategies.

This study’s robust methodology, leveraging large GWAS datasets and advanced statistical techniques, provides a comprehensive analysis while recognizing the limitations inherent to genetic studies, such as population-specific effects and environmental interactions not fully captured. Further research should consider these factors and explore the identified loci in diverse populations to enhance the generalizability of these findings.

## Abbreviations

AAC abdominal aortic calcium

CAC coronary artery calcium

DBP diastolic blood pressure

SBP systolic blood pressure

SNPs single nucleotide polymorphisms
